# Supplementary material for: How using brain-machine interfaces influences the human sense of agency
Source: PLoS One. 2021 Jan 7;16(1):e0245191. doi: 10.1371/journal.pone.0245191 (PMC7790430; doi:10.1371/journal.pone.0245191)
Supplement: Attachment — Submitted filename: BCI_Paper_ResponseToReviewers#3.docx [file pone.0245191.s001.docx]

Reviewer #2: Thank you for the revisions. I only have a few comments that should be addressed.

General Discussion (page 36, lines 22/24): You state that “[agency] did not differ between a condition in which participants used their own hand and a condition in which they used the robotic hand to perform a keypress”

This is not true as stated and should be removed/edited. As before, you should distinguish between the JoA and the FoA here. For the JoA, no comparison between the real hand and the BCI was made. So this statement is simply not supported by any data. One would have to use the same questionnaire for the real hand condition to determine if the JoA differed between the two conditions. Similarly, FoA (via delay estimates) was only compared in study 1, and there you have an interesting interaction between the factors delay and condition.

You could rephrase this by stating that participants report a high/strong JoA for BCIs (but leave out a comparison which was not part of the study design). FoA, in study 1, was comparable in the two conditions. (You could add a Bayesian test to see if there is evidence to “support the null”).

🡪 We have now reported the BF for this comparison, which indeed supported H0 (BF_10_=.207). We have added this result in the result section and we have also added a note regarding how this value was obtained using JAPS and its default priors.

🡪 In the general discussion section, we have modified the sentence as follow: “*We found in Study 1 that the absence of sensorimotor information was not detrimental for the feeling of agency, as measured through interval estimates, which did not differ between a condition in which participants used their own hand and a condition in which they used the robotic hand to perform a keypress.*”

Study 2 – Interval estimates. Please include the actual estimate results here, as for study 1. What are the means ±SDs and do participants distinguish between the levels? These will be relevant values to report for subsequent studies investigating Intentional Binding paradigms for BCIs/prostheses, too.

🡪 Participants who did not discriminate between levels were excluded, as reported in the participant section with the exclusion criteria. Out of 30 participants, 4 did not discriminate with a significant trend the three intervals. So, the 26 remaining participants for this experiment did significantly discriminate between the 3 delays.

🡪 We agree it could be interesting but it is not the same comparison as in Study 1 since here there was not real hand condition, so we did not run the same analysis than in Study 1. We have nonetheless added the means and SDs for the 3 intervals in the “interval estimates” section.

Page 15, ownership question – “I felt as if the robotic hand belonged to me”. (missing -ed)

🡪 This has been corrected

Page 27, line 20 – I am not sure that “volition” can be used in this way. Maybe something like a greater cognitive effort/more concentration etc. would be better.

🡪 We have replaced the word “volition” by “a greater cognitive effort”
